# Sequential motor learning transfers from real to virtual environment

**DOI:** 10.1186/s12984-021-00903-6

**Published:** 2021-06-30

**Authors:** Yuhi Takeo, Masayuki Hara, Yuna Shirakawa, Takashi Ikeda, Hisato Sugata

**Affiliations:** 1 Department of Rehabilitation, Oita University Hospital, Oita, Japan; 2 Graduate School of Welfare and Health Science, Oita University, Oita, Japan; 3 Graduate School of Science and Engineering, Saitama University, 255 Shimo-Okubo, Sakura-ku, 338-8570 Saitama City, Saitama Japan; 4 Faculty of Welfare and Health Science, Oita University, 700, Dannoharu, 870-1192 Oita, Japan; 5 Research Center for Child Mental Development, Kanazawa University, Kanazawa, Japan

**Keywords:** Sequential motor learning, Virtual environment, Real environment, Transfer of motor learning

## Abstract

**Background:**

Skill acquisition of motor learning between virtual environments (VEs) and real environments (REs) may be related. Although studies have previously examined the transfer of motor learning in VEs and REs through the same tasks, only a small number of studies have focused on studying the transfer of motor learning in VEs and REs by using different tasks. Thus, detailed effects of the transfer of motor skills between VEs and REs remain controversial. Here, we investigated the transfer of sequential motor learning between VEs and REs conditions.

**Methods:**

Twenty-seven healthy volunteers performed two types of sequential motor learning tasks; a visually cued button-press task in RE (RE task) and a virtual reaching task in VE (VE task). Participants were randomly assigned to two groups in the task order; the first group was RE task followed by VE task and the second group was VE task followed by RE task. Subsequently, the response time in RE task and VE task was compared between the two groups respectively.

**Results:**

The results showed that the sequential reaching task in VEs was facilitated after the sequential finger task in REs.

**Conclusions:**

These findings suggested that the sequential reaching task in VEs can be facilitated by a motor learning task comprising the same sequential finger task in REs, even when a different task is applied.

## Background

Motor learning refers to an improvement in the performance of sensory-guided motor behavior via practice [1]. The acquisition of new skills through motor practice is essential to interact with the environment and adjust the integration of multiple elements of movement. The two components of the learning process [2, 3] include implicit motor learning that improves the performance of a sequence without the knowledge of the sequence [4] and explicit motor learning that involves conscious recollection with the knowledge of the sequence. The memory system employed differs between implicit and explicit learning [5]. Explicit motor learning is associated with the activity of the dorsal premotor cortex, dorsolateral prefrontal cortex, and supplementary motor area [6]. By contrast, implicit learning is primarily related to the activity of the contralateral sensory and primary motor cortices [7]. Previous studies focusing on implicit motor learning have reported the activations of the right ventral striatum [8–10], right thalamus [9], and subcortical regions [11] during a serial reaction time task (SRTT). This implicit sequential task has been widely used to quantitatively evaluate the acquisition of a new skill [12–14].

Virtual environments (VEs) have attracted attention with the recent advances in technology. VEs can modulate perception and cognition by providing coherent sensory feedback that corresponds to the actions taken place within the VE and grants users the psychological sense of being present there [15]. To illustrate, previous studies have reported that both the agency and ownership of the virtual hand are induced concurrently if the visual presentation of the virtual hand movement matches the subject’s active hand movement [16–18, 19]. Due to their applicability and expandability, VEs have been introduced as new effective approaches in rehabilitation. For instance, previous studies have demonstrated that VEs could contribute to effective interventions for the improvement of upper limb function, balance, and gait after stroke [20–23]. Other studies have suggested that VE training could improve balance, gait, motor function, and quality of life in patients with Parkinson’s disease [24–26] and motor function in children with cerebral palsy [27]. Furthermore, several studies have reported that VEs also promote motor learning [9, 15, 16]. However, opinions vary concerning how learning in virtual and real environments (REs) could affect motor skill acquisition. For example, a previous study has suggested that post-training performance in virtual and real training is equivalent and both of which significantly exceeds without training [28]. However, another study demonstrated that training in VE cannot promote better performance than the same task in RE [29]. Furthermore, several reports focused on interactions of skill acquisition of motor learning in VEs and REs. For instance, a study in healthy subjects demonstrated that skill acquisition of the sequential motor learning occurs at the same rate in both VE and conventional screen environments, while the transfer of motor skills was not observed from VE to the screen environment [30]. Another study reported that skill acquisition in individuals with the neuromuscular disease transferred from VEs to REs [31]. In contrast, motor learning and motor performance did not transfer from VEs to REs in older adults and individuals with neuromuscular diseases [29, 32, 33]. These results imply the presence of some relationship in skill acquisition of motor learning between VEs and REs. However, whether the transfer of skill acquisition of motor skills occurs between VEs and REs is still controversial. The above-mentioned studies investigated the relationship between motor learning with the same tasks between different environments (VE and RE), whereas few studies focused on motor learning with different tasks in different environments. As for motor learning transfer, previous studies also suggested motor skill learning transfer in the case of the same environments concerning different tasks. For instance, the transfer of partial learning has been reported using SRTT [34], and motor skill transfer from one hand to the other has also been suggested [35]. Furthermore, motor skill transfer from one hand to the other was demonstrated in VEs [36]. Considering the relationship between motor learning skill acquisition between VEs and REs, we hypothesized that motor learning transfer occurs between VEs and REs even in the case of different tasks.

Therefore, the present study was aimed at revealing whether motor learning transfer could occur between VEs and REs even in the case of different tasks. In the present study, participants were randomly assigned to one of two groups. Both groups performed sequential motor learning tasks in both environments. However, one group performed a sequential button-press task in RE first, followed by a sequential reaching task in VE. The second group performed a sequential reaching task in VE, followed by a sequential button-press task in RE. To examine motor learning transfer, we compared the response time between the two motor learning tasks.

## Methods

### Participants

Thirty healthy volunteers (21.5 ± 3.0 years; 23 women) participated in this study. All participants were right-handed, as determined by the Edinburgh Handedness Inventory Test (Oldfield, 1971). No participant had a history of neurological or psychiatric disease, and all had a normal or corrected-to-normal vision (10 participants wore contact lenses). In accordance with the Declaration of Helsinki, we explained the purpose and possible consequences of this study to all participants and obtained their informed consent before the study commenced.

### Experimental design

**Fig. 1:**
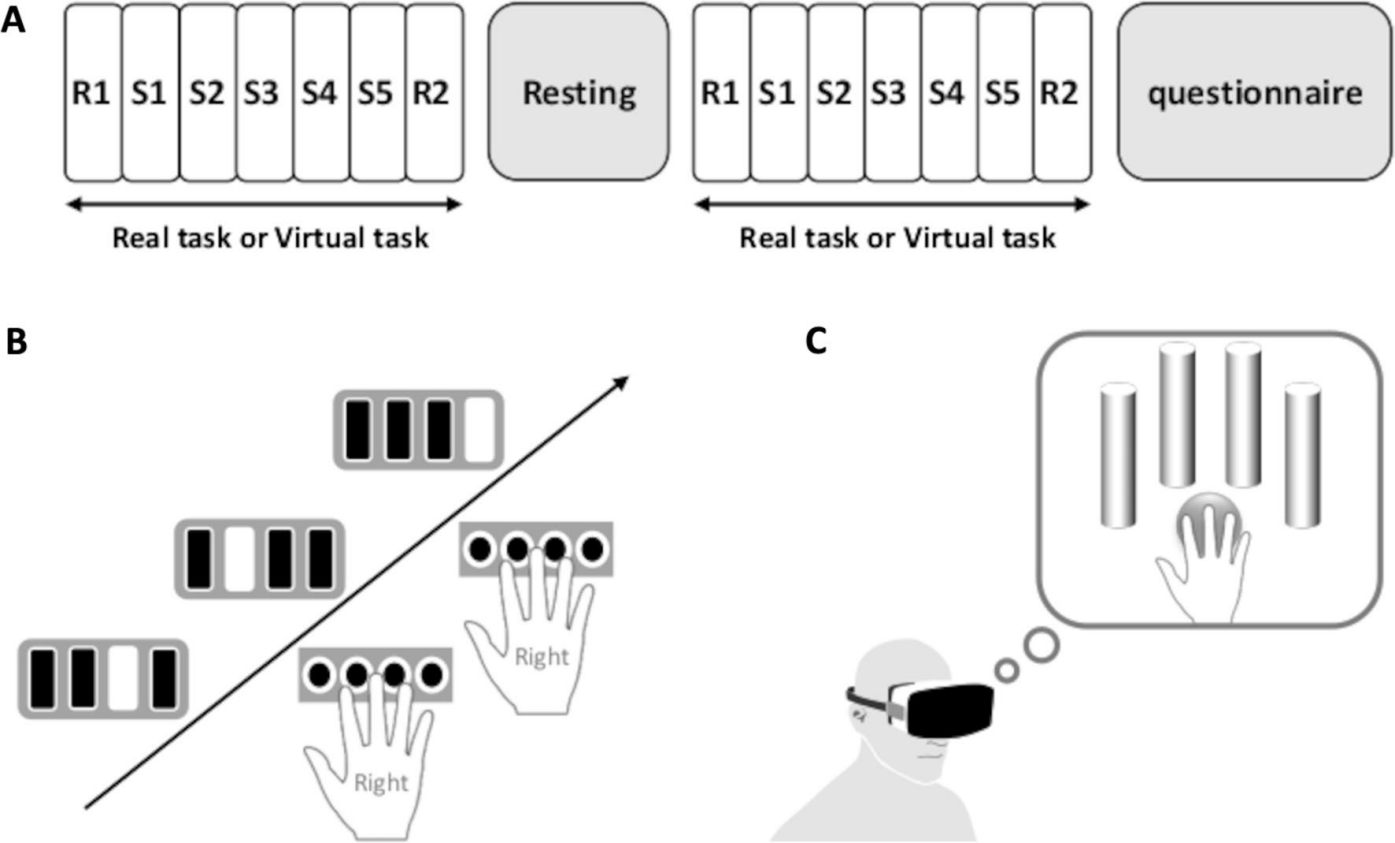
Experimental procedure. **A** The participants performed sequential motor learning tasks in both real environments (REs); a visually cued button-press task and virtual environments (VEs); a virtual reaching task. After completing all experiments, the participants answered a questionnaire regarding virtual hand illusions. Interval between the tasks was 5-minutes. Task order was randomly and evenly distributed. **B** In RE task, participants performed a 12-digit visually cued button-press sequence task consisting of six blocks (S1–S6) and two random blocks (R1, R2). Random blocks were set before (R1) and after (R2) sequence blocks. **C** In the VE task, participants performed a 12-digit virtual reaching sequence task consisting of six blocks (S1-S6) and two random blocks (R1, R2). Random blocks were set before (R1) and after (R2) sequence blocks. Sequential motor learning tasks in both REs and VEs were composed by a 12-digit same sequence

In order to examine the transfer of motor learning, the participants performed sequential motor learning tasks in both REs and VEs (Fig. 1A). For the motor learning task in a RE, the participants performed a visually cued button-press task (RE task; Fig. 1B). For the motor learning task in a VE, the participants performed a virtual reaching task (VE task; Fig. 1C).

The participants were randomly assigned to two groups to keep a counter balance in the task order; the first group was RE task followed by VE task (RE-VE group) and the second group was VE task followed by RE task (VE-RE group). There was a 5-minute interval between the tasks. After completing all experiments, the participants were asked to answer a virtual hand illusion (VHI) questionnaire. Considering fatigue, the total time of the performed tasks was controlled in both groups (RE task: RE-VE group 570.2 ± 27.0 s, VE-RE group 569.2 ± 27.1 s, VE task: RE-VE group 890.0 ± 143.3 s, VE-RE task 896.9 ± 241.7 s). Three participants (one in the RE-VE group and two in the VE-RE group) were excluded from data analysis as their mean responses were beyond ± 2SD from the mean for subjects. Thus, the final sample size included 27 participants, of which 13 were in the RE-VE group and 14 in the VE-RE group.

### Motor learning task in RE (RE task)

**Fig. 2:**
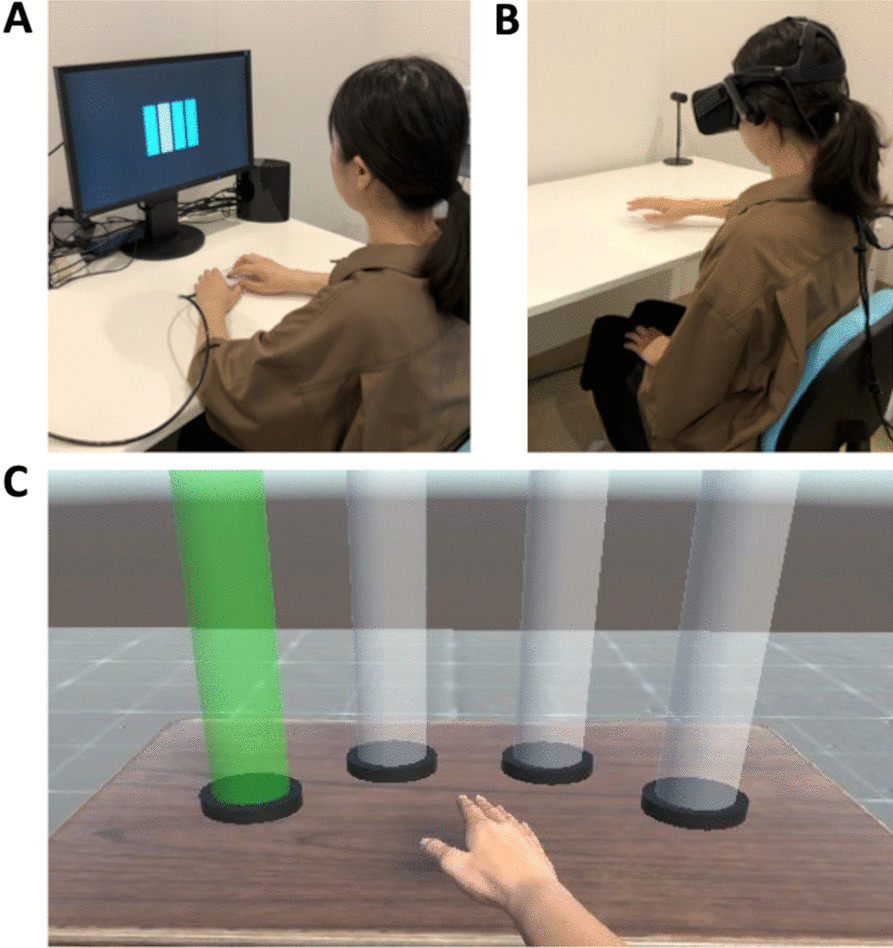
Experimental environments. **A** Real environment; Four horizontal bars were displayed on the screen. When the color of a bar changed from gray to blue, the participants were instructed to press the corresponding button as quickly and accurately as possible. **B** The participant wore a head-mounted display and sat in a chair at a specific position where they could reach all the targets and the base point easily with minimal arm movement. **C** Virtual environment; When participants touched the base point with their right virtual hand, the color of target changed from gray to green. The participants were instructed to reach the target as quickly and accurately as possible

The participants performed a visually cued button-press task consisting of a 12-digit motor sequential learning task in which participants were required to react with their four right-hand fingers (Fig. 1A) [14]. Four horizontal bars were displayed on the screen (Fig. 2A). When the color of a bar changed from gray to blue, the participants were instructed to press the corresponding button as quickly and accurately as possible. If a participant pressed the correct button, the next stimulation was presented after 1 s. If a participant pressed an incorrect button, the stimulation was unchanged until the participant pressed the correct button. This motor learning task consisted of five sequence blocks (S1–S5) and two random blocks (R1, R2). Random blocks were set before (R1) and after the five sequence blocks (R2). The sequence blocks comprised five repeats of 12 stimuli in the same sequence. Thus, a total of 300 button presses were performed in a sequence block, whereas 120 button presses were performed in a random block.

### Motor learning task in VE (VE task)

In the motor learning task in VE, four transparent gray targets with a cylindrical shape and a transparent blue base point with a hemispherical shape were displayed on a virtual desk located in a VE. The participant was fixed with a head-mounted display (HMD) and instructed to sit in a chair at a specific position where they could reach all the targets and the base point easily with minimal arm movement, and to perform a virtual reaching task (Fig. 2B). The base point disappeared when the participants kept their virtual hand in the hemisphere for 1 s, which meant the beginning of an experimental trial. As the color of a target changed from gray to green, the participants were instructed to put the virtual right hand, which was rendered overlapping at the participant’s hand position in the RE, into the designated target as quickly and accurately as possible. The color of target changed from green to red when the participants reached the target. As the base point was displayed again just after the virtual hand successfully reached the target; the participants could start the next trial whenever they liked by touching it. However, if the virtual hand could not reach the intended target within 2 s, the color of the target changed from green to red together with the re-emergence of the base point. In this case, the color of the target returned to gray when the participant touched the base point, and the next trial was presented.

To examine the transfer of sequential motor learning, the orders of sequence were exactly same in both tasks. Thus, VE task also consisted of five sequence blocks (S1–S5) and two random blocks (R1, R2) and a total of 300 virtual reaching were performed in a sequence block, whereas 120 virtual reaching were performed in a random block.

### Apparatuses

In the RE task, visual stimuli were applied by Presentation System (Neurobehavioral Systems, USA) and recording of response times were realized by fiber optic computer response system (PKG-9904, Current Design Inc., USA) (Fig. 2A).

In the VE task, we developed a Virtual Reality experimental system by integrating an HMD (Oculus Rift, Oculus) and a hand tracking system (Leap Motion, Ultraleap Ltd.) into a game engine (Unity 2018.4.24, Unity Technologies) (Fig. 2B). Our VE task mainly consisted of four cylindrical targets, a hemispherical base point, a desk, and a virtual right hand. Leap Motion attached on the HMD tracked the participant’s hand position, orientation, and posture, and the information was applied to render the virtual hand in the VE. The four cylindrical targets were symmetrically allocated every 30 degrees centering around the base point (Fig. 2B); the distance between the base point and each target were 25 cm in the scale of RE. The collision detection with the targets or base point was based on the distance between their center positions and the palm position of virtual hand. It was considered that the hand reached a target or base point when the distance became smaller than their diameters; in the scale of RE, the diameters of targets and base point were 10 cm, respectively. Rendering of VE was performed in 80 Hz which was the same as the refresh rate of Oculus Rift. The sampling time for the experiment control was set in 20 ms (i.e., 50 Hz sampling rate), within which the collision detection, control of experimental condition, and data acquisition were performed.

**Table 1:** Virtual hand illusion questionnaire (VHI questionnaire)

Item	Assertion
Embodiment	
Q1	I felt as if the virtual hand was my own hand.
Sense of agency	
Q2	The movement of virtual hand matched with the movement produced by my hand.
Control	
Q3	I felt like my hand was becoming bigger.
Q4	I could not feel my hand.
Q5	I felt as if my hand was turning “virtual.”
Q6	I felt as if my whole hand was moving.

### VHI questionnaire

We subjectively evaluated the participant’s experience or feeling of the right hand during movements of the virtual right hand. The participants were asked to answer a VHI questionnaire with a seven-point Likert scale (− 3 to + 3). In the seven-point Likert scale, − 3 and + 3 were set as “I strongly disagree with the statement” and “I strongly agree with the statement”, respectively; 0 was considered as a neutral rating allocated for unjudgeable experience. The applied questionnaire items, based on the original rubber hand illusion questionnaire [37], are shown in Table 1.

The first two items were designed to correspond to the VHI. The illusion items assessed the embodiment of virtual hand (Q1) and the sense of agency (Q2) during the experiment, respectively. The other items served as control for suggestibility, which were unrelated to the VHI. The suggestibility meant that sometimes the participants rated all the items in the same manner for any reason and was therefore removed from the analysis.

### Statistical analysis

Statistical analyses were performed using MATLAB (R2017a) and SPSS (version 25). First, the effects of time (R1 vs. S5) and task order (RE-VE group vs. VE-RE group) on the response times were assessed with a two-way repeated measure analysis of variance (ANOVA). The Greenhouse–Geisser correction was applied to the degrees of freedom when the sphericity assumption was violated. In case of significant effects, post hoc analyses were performed to test interaction effects with unpaired *t*-tests. Next, the mean rating for Q1 and Q2 was compared with RE-VE group and VE-RE group using a Mann–Whitney U test.

## Results

### Response time in the RE task

**Fig. 3:**
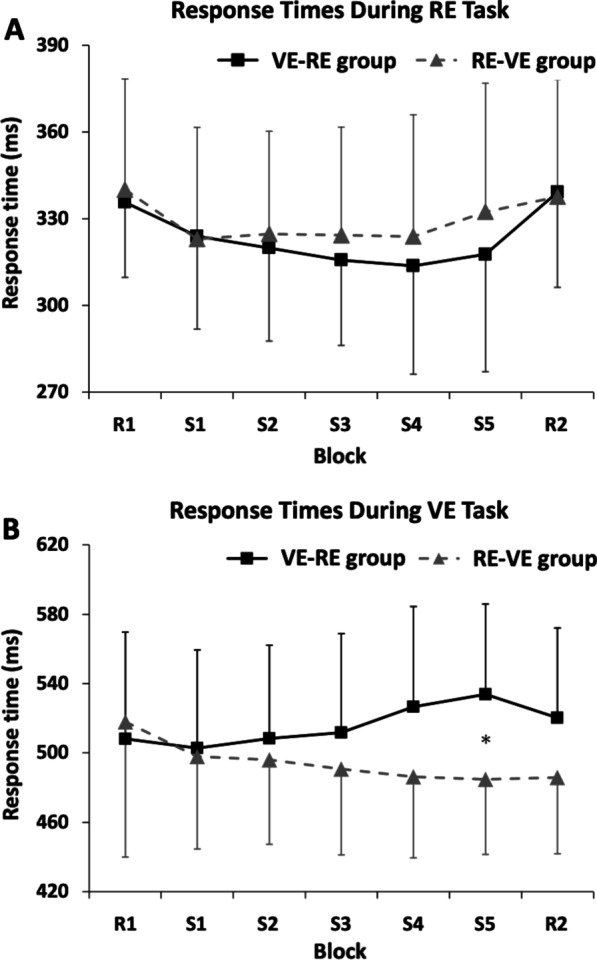
Response times during the RE and VE tasks. **A** Response times during the RE task. During the RE task, we observed a significant main effect of *time* (Blocks) (*p* < 0.05). **B** Response times during the VE task. During the VE task, we observed no significant main effect of *time* (Blocks), although we could obtain a significant *time* × *task order* (RE-VE group vs. VE-RE group) interaction (*p* < 0.05). Post hoc analyses revealed that response time was significantly different between two groups only in S5 block (*p < 0.05)

A two-way repeated measure ANOVA with factors *time* (Blocks) and *task order* (RE-VE group vs. VE-RE group) showed a significant main effect of *time* (*F*_(6,25)_ = 5.121, *p* = 0.003, *η*^*2*^*p* = 0.17) but not *task order* (*F*_(1,25)_ = 0.197, *p* = 0.661, *η*^*2*^ *p* = 0.008) or *time* × *task order* interaction (*F*_(6,25)_ = 0.667, *p* = 0.568, *η*^*2*^ *p* = 0.026) (Fig. 3A).

### Response time in the VE task

A two-way repeated measure ANOVA with factors *time* (Blocks) and *task order* (RE-VE group vs. VE-RE group) revealed no significant main effect of time (*F*_(6,25)_ = 0.984, *p* = 0.387, *η*^*2*^ *p* = 0.038) and task order (*F*_(1,25)_ = 1.184, *p* = 0.287, *η*^*2*^ *p* = 0.045), and a significant *time* × *task order* interaction (*F*_(6,25)_ = 4.835, *p* = 0.01, *η*^*2*^ *p* = 0.162) (Fig. 3B). In order to further investigate the significant *time* × *task order* interaction, post hoc analyses were performed for each block with Student *t*-test. The results showed significant differences between the RE-VE and VE-RE groups only in the S5 block (*t*_(25)_ = 2.551, *p* = 0.017).

### VHI questionnaire

**Fig. 4:**
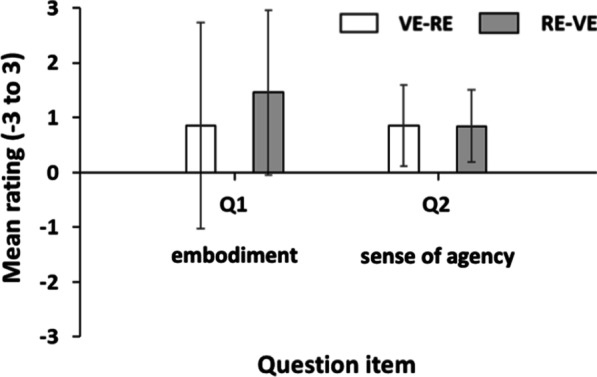
Results of virtual hand illusion questionnaire about Q1 and Q2 between VE-RE group and RE-VE group. No significant difference between VE-RE group and RE-VE group was observed

A Mann–Whitney U test showed no significant difference in VHI questionnaire between VE-RE group and RE-VE group (Q1: *z* = − 0.351, *p* = 0.725; Q2: *z* = − 0.30, *p* = 0.764) (Fig. 4).

## Discussion

The present study investigated the transfer of motor learning between VEs and REs in different tasks. The results suggested that motor learning in VEs may be facilitated after motor learning in REs even if different tasks are applied.

VEs enable individuals to have virtual experiences that are similar to those in REs. However recent studies have shown that the learning mechanism between VEs and REs is different [38]. As for the neurophysiological aspects of visual processes, visual information is processed thorough ventral and dorsal pathways. The ventral path provides perception and identification of visual inputs, and the dorsal path monitors real-time information [39, 40]. In the REs, the visual information within peripersonal space was processed using the dorsal path [41], whereas visual processing in VEs induced the ventral path [42]. These results indicate that processing of sensory information in the brain between VEs and REs is different. In the present study, motor learning in REs improved motor learning in VEs, whereas motor learning in VEs did not improve motor learning in REs. Some studies have demonstrated that motor learning in VEs requires more brain activity than in REs for cognitive and motor control [43, 44]. In addition, Ranganathan et al. [45] reported that motor learning in different environments interferes with each other. If the first task does not share task environments with the later task, there is prolonged interference in learning the later task. However, another study reported that motor learning in REs involved more implicit motor learning compared with motor learning in VEs [46]. In addition, studies on motor learning transfer in REs with different tasks have suggested that the higher the similarity of tasks, the higher is the transfer [47, 48]. Moreover, previous studies have demonstrated that the transfer of implicit motor learning is not observed in the cases of changing response locations [49] or stimulus–response associations [50]. Furthermore, different tasks have been shown to cause interference or inhibit the transfer of motor learning [51, 52]. Given these findings, we would not expect the transfer of motor learning between the sequential button-press task and sequential reaching task to be induced in REs. Thus, these results support our finding that sequential motor learning in VEs is facilitated after sequential motor learning in REs despite interference with different environments and tasks. However, additional research is required to assess this possibility because we did not evaluate neurophysiological changes using electroencephalogram or functional magnetic resonance imaging.

In the present study, the virtual reaching task in VEs did not promote sequential learning in the VE-RE group. Previous research on VEs employed several tools, such as a two-joint mechanical arm [53], joysticks [54] and pinch force sensors [30], which showed improvement in the motor learning ability. These experiments were performed using sensory information as clues instead of visual information, like the arm-reaching task used in the present study. Another study reported that providing tactile feedback using sensor gloves in VEs reduces the discrepancy between the virtual and physical environments [55]. These results indicate that the arm-reaching task in VEs may not facilitate sequential learning because of the lack of tactile stimulation. Furthermore, several studies have reported that motor learning in VEs is influenced by individual factors [30, 56, 57]. For instance, experience with video games showed a positive impact on performance in VEs [30, 58]. Considering the reports of the aforementioned studies, the lack of motor learning capacity in VEs observed in the present study may be attributed to individual factors.

The present study has several limitations. First, although the present study focused on motor learning transfer with different tasks in different environments, motor learning transfer was investigated only between a visual button-press task in RE and a virtual reaching task in VE. Therefore, we could not rule out the impact of task-dependent (button-press task in VE versus arm-reaching task in RE) and environment-dependent (button-press task in RE versus button-press task in VE, arm-reaching task in RE versus arm-reaching task in VE) factors. Also, we could not deny the effects of environmental differences with wearing the HMD since we did not wear the HMD in REs. However, to the best of our knowledge, no study has been published that focuses on motor learning transfer with different tasks in different environments. Therefore, we believe that our findings could trigger the expansion of VE studies focusing on motor learning transfer of different variables, such as tasks and environments. Second, in the present study, the effects of motor learning were evaluated relative to the response time alone. The results of other motor learning studies could be different due to study design, variations of the tasks, methodological differences, and the phase of motor learning [53]. For example, a motor learning study focusing on visuomotor adaptation described investigations based on the movement trajectory and angles as variables [54], suggesting that the present results might represent only parts of the puzzle in the field of motor learning. According to the type of assessed motor learning, the appropriate variables related to motor learning need to be carefully selected. Third, we subjectively evaluated the experience of the participants or their feeling of the right hand during the movements of the virtual right hand using the VHI questionnaire [22]. Interestingly, recent studies also propose further questionnaires that include multiple items of embodiment and sense of agency [59, 60]. These questionnaires might also be used to provide further support. Finally, we used the SRTT proposed by Nissen and Bullemer to assess implicit learning [14]. However, time constraints did not allow us to test offline learning as a consolidation effect. Therefore, we could not deny the possibility that it was the performance with short-term change instead of learning with long-term change. Future studies would be required, spanning over separate days, to assess memory retention.

## Conclusions

The present study investigated whether the transfer of motor learning can occur even in different tasks between VEs and REs. In order to examine the transfer of motor learning, we compared the response time between both motor learning tasks. Results showed that the sequential reaching task in VEs was facilitated after the sequential finger task in REs. These results suggested that motor learning in VEs may be facilitated after motor learning in REs despite interference with different environments and tasks. However, we cannot discuss the neurophysiological aspect for the transfer of motor learning between VEs and REs since we did not evaluate the neurophysiological data. Thus, to demonstrate the detailed neural mechanism of sequential learning from RE to VE, further study is needed.

## Data Availability

The datasets used and/or analyzed during the current study are available from the corresponding author on reasonable request.

## Abbreviations

VEs: virtual environments.
REs: real environments.
SRTT: serial reaction time task.
VHI: virtual hand illusion.
HMD: head-mounted display.
